# Supplementary material for: Herpes zoster epidemiology in Latin America: A systematic review and meta-analysis
Source: PLoS One. 2021 Aug 12;16(8):e0255877. doi: 10.1371/journal.pone.0255877 (PMC8360515; doi:10.1371/journal.pone.0255877)
Supplement: S1 File — (DOCX) [file pone.0255877.s002.docx]

# Supporting information

## S1 Text. Database search strategy.

PubMed 03-03-2020

| **Search** | **Query** |
| --- | --- |
| #16 | Search (#13 AND #14) Filters: Publication date from 2000/01/01 |
| #15 | Search (#13 AND #14) |
| #14 | Search ((Americas[MeSH Terms:noexp] OR America*[tiab] OR Latin America[Mesh] OR Latin America*[tiab] OR Latinamerica*[tiab] OR Latinoamerica*[tiab] OR Latin*[tiab] OR Hispanic Americans[Mesh] OR Hispanic America*[tiab] OR Hispanoamerica*[tiab] OR Iberoamerica*[tiab] OR Ibero Americ*[tiab] OR Panamerican*[tiab] OR Central America[Mesh] OR Central America*[tiab] OR Centroamerica*[tiab] OR Mesoamerica*[tiab] OR Meso America*[tiab] OR Middle America*[tiab] OR South America[Mesh] OR South America*[tiab] OR Southamerica*[tiab] OR Sudamerica*[tiab] OR “America del sur”[tiab] OR Caribbean Region[Mesh] OR Caribbean[tiab] OR Caribe*[tiab] OR West Indies[Mesh] OR West Indi*[tiab] OR Antill*[tiab] OR Indians, South American[Mesh] OR Indians, Central American[Mesh] OR Amerindian*[tiab] OR Indians[tiab] OR American Indian*[tiab] OR Native America*[tiab] OR Patagoni*[tiab] OR Andes[tiab] OR Andean*[tiab] OR Amazon*[tiab] OR Argentin*[ad] OR Argentin*[tiab] OR Argentina[pl] OR Bolivia*[ad] OR Bolivia*[tiab] OR Bolivia[pl] OR Brazil*[ad] OR Brazil*[ad] OR Brazil*[tiab] OR Brazil*[tiab] OR Brazil[pl] OR Colombia*[ad] OR Colombia*[tiab] OR Colombia[pl] OR Chile*[ad] OR Chile*[tiab] OR Chile[pl] OR Ecuador*[ad] OR Ecuator*[ad] OR Ecuador*[tiab] OR Ecuador[pl] OR Guiana*[ad] OR Guiana*[tiab] OR French Guiana[pl] OR Guyan*[ad] OR Guyan*[tiab] OR Guyana[pl] OR Paraguay*[ad] OR Paraguay*[tiab] OR Paraguay[pl] OR Peru*[ad] OR Peru*[tiab] OR Peru[pl] OR Surinam*[ad] OR Surinam*[tiab] OR Suriname[pl] OR Uruguay*[ad] OR Uruguay*[tiab] OR Uruguay[pl] OR Venez*[ad] OR Venez*[tiab] OR Venezuela[pl] OR Belize*[ad] OR Belize*[tiab] OR Belize[pl] OR Costa Ric*[ad] OR Costarric*[ad] OR Costaric*[ad] OR Costa Ric*[tiab] OR Costarric*[tiab] OR Costaric*[tiab] OR Costa Rica[pl] OR Salvador*[ad] OR Salvador*[tiab] OR El Salvador[pl] OR Guatemal*[ad] OR Guatemal*[tiab] OR Guatemala[pl] OR Hondur*[ad] OR Hondur*[tiab] OR Honduras[pl] OR Nicaragu*[ad] OR Nicaragu*[tiab] OR Nicaragua[pl] OR Panam*[ad] OR Panam*[tiab] OR Panama[pl] OR Mexico[Mesh] OR Mexic*[ad] OR Mexic*[tiab] OR Mejic*[tiab] OR Mexico[pl] OR Cuba*[ad] OR Cuba*[tiab] OR Cuba[pl] OR OR Dominic*[ad] OR Dominic*[tiab] OR Dominican Republic[pl] OR Haiti*[ad] OR Haiti*[tiab] OR Haiti[pl] OR Jamaic*[ad] OR Jamaic*[tiab] OR Jamaica[pl] OR Puerto Ric*[tiab] OR Puertorric*[tiab] OR Puertoric*[tiab])) |
| #13 | Search (#1 OR #2 OR #3 OR #4 OR #5 OR #6 OR #7 OR #8 OR #9 OR #10 OR #11 OR #12) |
| #12 | Search Culebrilla[tiab] |
| #11 | Search Post-Herpetic[tiab] |
| #10 | Search Postherpetic[tiab] |
| #9 | Search Neuralgia, Postherpetic[Mesh] |
| #8 | Search Herpes-Virus 3[tiab] |
| #7 | Search HHV3[tiab] |
| #6 | Search Shingles[tiab] |
| #5 | Search Herpesvirus 3[tiab] |
| #4 | Search VZ[tiab] |
| #3 | Search Herpesvirus 3, Human[Mesh] |
| #2 | Search Zoster[tiab] |
| #1 | Search Herpes Zoster[Mesh] |

**EMBase (Elsevier) 03-03-2020**

| **No.** | **Query Results** |
| --- | --- |
|  |  |
| #16 #15 | AND (2000:py OR 2001:py OR 2002:py OR 2003:py  OR 2004:py OR 2005:py OR 2006:py OR 2007:py OR  2008:py OR 2009:py OR 2010:py OR 2011:py OR  2012:py OR 2013:py OR 2014:py OR 2015:py OR  2016:py OR 2017:py OR 2018:py OR 2019:py OR  2020:py) |
| #15 #13 AND #14 | |
| #14 | americas:ti,ab OR 'south and central america'/exp  OR ((latin NEAR/1 america*):ti,ab) OR  latinamerica*:ti,ab OR latinoamerica*:ti,ab OR  hispanoamerica:ti,ab OR iberoamerica*:ti,ab OR  ((ibero NEAR/1 americ*):ti,ab) OR  panamerica*:ti,ab OR ((south NEAR/1  america*):ti,ab) OR southamerica*:ti,ab OR  sudamerica*:ti,ab OR (america:ti,ab AND del:ti,ab  AND sur:ti,ab) OR ((central NEAR/1  america*):ti,ab) OR centroamerica*:ti,ab OR  mesoamerica*:ti,ab OR ((meso NEAR/1  america*):ti,ab) OR ((middle NEAR/1  america*):ti,ab) OR 'caribbean'/exp OR 'caribbean  islands'/exp OR caribbean*:ti,ab OR caribe*:ti,ab  OR ((west NEAR/1 indi*):ti,ab) OR antill*:ti,ab  OR 'american indian'/exp OR amerindian*:ti,ab OR  indians:ti,ab OR ((native NEAR/1 america*):ti,ab)  OR patagoni*:ti,ab OR andes:ti,ab OR  andean*:ti,ab OR amazon*:ti,ab OR 'argentina'/exp  OR argentin*:ti,ab OR 'bolivia'/exp OR  bolivia*:ti,ab OR 'brazil'/exp OR brazil*:ti,ab  OR brazil*:ti,ab OR 'colombia'/exp OR  colombia*:ti,ab OR 'chile'/exp OR chile*:ti,ab OR  'ecuador'/exp OR ecuador*:ti,ab OR 'french  guiana'/exp OR guiana*:ti,ab OR 'guyana'/exp OR  guyan*:ti,ab OR 'paraguay'/exp OR paraguay*:ti,ab  OR 'peru'/exp OR peru*:ti,ab OR 'suriname'/exp OR  surinam*:ti,ab OR 'uruguay'/exp OR uruguay*:ti,ab  OR 'venezuela'/exp OR venez*:ti,ab OR  'belize'/exp OR beliz*:ti,ab OR 'costa rica'/exp  OR 'costa rica':ti,ab OR costarric*:ti,ab OR  costaric*:ti,ab OR 'el salvador'/exp OR  salvador*:ti,ab OR 'guatemala'/exp OR  guatemal*:ti,ab OR 'honduras'/exp OR  hondur*:ti,ab OR 'nicaragua'/exp OR  nicaragu*:ti,ab OR 'panama'/exp OR panam*:ti,ab  OR 'mexico'/exp OR mexic*:ti,ab OR mejic*:ti,ab  OR 'cuba'/exp OR cuba*:ti,ab OR 'dominican  republic'/exp OR dominica*:ti,ab OR 'haiti'/exp  OR haiti*:ti,ab OR 'jamaica'/exp OR jamaic*:ti,ab  OR 'puerto rico'/exp OR ((puerto NEAR/1  ric*):ti,ab) OR puertoric*:ti,ab OR  puertorric*:ti,ab |
| #13. #1 OR #2 OR #3 OR #4 OR #5 OR #6 OR #7 OR #8 OR #9 OR #10 OR #11 OR #12 | |
| #12 | culebrilla:ti,ab |
| #11 | 'post herpetic':ti,ab |
| #10 | postherpetic:ti,ab |
| #9 | 'postherpetic neuralgia'/exp |
| #8 | 'herpes-virus 3':ti,ab |
| #7 | hhv3:ti,ab |
| #6 | shingles:ti,ab |
| #5 | 'herpesvirus 3':ti,ab |
| #4 | vz:ti,ab |
| #3 | 'varicella zoster virus'/exp |
| #2 | zoster:ti,ab |
| #1 | 'herpes zoster'/exp |

LILACS (BVS-EN) 03-03-2020

| MH Herpes Zoster OR Zoster OR MH Herpesvirus 3, Human OR VZ OR Shingles OR HHV3 OR Cobreiro OR Cobrelo OR Culebrilla OR Culebrón OR MH Neuralgia, Postherpetic OR Postherpetic$ OR Pós-Herpétic$ OR Póst-Herpétic$ [Words] and 2000 OR 2001 OR 2002 OR 2003 OR 2004 OR 2005 OR 2006 OR 2007 OR 2008 OR 2009 OR 2010 OR 2011 OR 2012 OR 2013 OR 2014 OR 2015 OR 2016 OR 2017 OR 2018 OR 2019 OR 2020  [Country, year publication] |
| --- |

**Cochrane Library (Wiley) 03-03-2020**

| **ID** | **Search** |
| --- | --- |
| #1 | MeSH descriptor: [Herpes Zoster] explode all trees |
| #2 | Zoster:ti,ab,kw |
| #3 | MeSH descriptor: [Herpesvirus 3, Human] explode all trees |
| #4 | VZ:ti,ab,kw |
| #5 | (Herpesvirus NEAR/1 3):ti,ab,kw |
| #6 | Shingles:ti,ab,kw |
| #7 | HHV3:ti,ab,kw |
| #8 | (Herpes-Virus NEAR/1 3):ti,ab,kw |
| #9 | MeSH descriptor: [Neuralgia, Postherpetic] explode all trees |
| #10 | Postherpetic:ti,ab,kw |
| #11 | Post-Herpetic:ti,ab,kw |
| #12 | Culebrilla:ti,ab,kw |
| #13 #1 OR #2 OR #3 OR #4 OR #5 OR #6 OR #7 OR #8 OR #9 OR #10 OR #11 OR #12 | |
| #14 | MeSH descriptor: [Americas] this term only |
| #15 | MeSH descriptor: [Latin America] explode all trees |
| #16 | MeSH descriptor: [Central America] explode all trees |
| #17 | MeSH descriptor: [South America] explode all trees |
| #18 | MeSH descriptor: [Caribbean Region] explode all trees |
| #19 | MeSH descriptor: [West Indies] explode all trees |
| #20 | MeSH descriptor: [Indians, South American] explode all trees |
| #21 | MeSH descriptor: [Indians, Central American] explode all trees |
| #22 | MeSH descriptor: [Mexico] explode all trees |
| #23 | MeSH descriptor: [Puerto Rico] explode all trees |
| #24 | ((Latin NEAR/1 America*) OR Latinamerica* OR Latinoamerica* OR Latin* OR Hispanic Americans OR Iberoamerica* OR (Ibero NEAR/1 Americ*) OR Panamerican* OR (Central NEAR/1 America*) OR Centroamerica* OR Mesoamerica* OR (Meso NEAR/1 America*) OR (Middle NEAR/1 America*) OR (South NEAR/1 America*) OR Southamerica* OR Sudamerica* OR (America NEAR/1 Sur) OR Caribbean OR Caribe* OR (West NEAR/1 Indi*) OR Antill* OR Amerindian* OR Indians OR (American NEAR/1 |

**CINAHL (EBSCO) 3-03-2020**

| **#** | **Query** |
| --- | --- |
| S18 | S13 AND S16  Limiters - Published Date: 20000101-20191231 |
| S17 | S13 AND S16 |
| S16 | S14 OR S15 |
| S15 | AB Latin America* OR Latinamerica* OR Latinoamerica* OR Latin* OR Hispanic Americans OR Iberoamerica* OR Ibero Americ* OR Panamerican* OR Central America* OR Centroamerica* OR Mesoamerica* OR Meso America* OR Middle America* OR South America* OR Southamerica* OR Sudamerica* OR America del sur OR Caribbean OR Caribe* OR West Indi* OR Antill* OR Amerindian* OR Indians OR American Indian* OR Native America* OR Patagoni* OR Andes OR Andean* OR Amazon* OR Argentin* OR Bolivia* OR Brazil* OR Brazil* Colombia* OR Colombia* OR Colombia OR Chile* OR Ecuador* OR Guiana* OR Guyan* OR Guyan* OR Paraguay* OR Paraguay* OR Peru* OR Surinam* OR Surinam* OR Uruguay* OR Venez* OR Belize* OR Costa Ric* OR Costarric* OR Costaric* OR Costa Ric* OR Costarric* OR Salvador* OR Salvador* OR El Salvador OR Guatemal* OR Guatemal* OR Guatemala OR Hondur* OR Nicaragu* Panam* OR Mexic* OR Cuba* OR Dominic* OR Dominic* OR Haiti* OR Jamaic* OR Puerto Ric* OR Puertorric* OR Puertoric* |
| S14 | TI Latin America* OR Latinamerica* OR Latinoamerica* OR Latin* OR Hispanic Americans OR Iberoamerica* OR Ibero Americ* OR Panamerican* OR Central America* OR Centroamerica* OR Mesoamerica* OR Meso America* OR Middle America* OR South America* OR Southamerica* OR Sudamerica* OR America del sur OR Caribbean OR Caribe* OR West Indi* OR Antill* OR Amerindian* OR Indians OR American Indian* OR Native America* OR Patagoni* OR Andes OR Andean* OR Amazon* OR Argentin* OR Bolivia* OR Brazil* OR Brazil* Colombia* OR Colombia* OR Colombia OR Chile* OR Ecuador* OR Guiana* OR Guyan* OR Guyan* OR Paraguay* OR Paraguay* OR Peru* OR Surinam* OR Surinam* OR Uruguay* OR Venez* OR Belize* OR Costa Ric* OR Costarric* OR Costaric* OR Costa Ric* OR Costarric* OR Salvador* OR Salvador* OR El Salvador OR Guatemal* OR Guatemal* OR Guatemala OR Hondur* OR Nicaragu* Panam* OR Mexic* OR Cuba* OR Dominic* OR Dominic* OR Haiti* OR Jamaic* OR Puerto Ric* OR Puertorric* OR Puertoric* |
| S13 | S1 OR S2 OR S3 OR S4 OR S5 OR S6 OR S7 OR S8 OR S9 OR S10 OR S11 OR S12 |
| S12 | TI Culebrilla OR AB Culebrilla |
| S11 | TI Post-Herpetic OR AB Post-Herpetic |
| S10 | TI Postherpetic OR AB Postherpetic |
| S9 | (MH "Neuralgia, Postherpetic") |
| S8 | TI Herpes-Virus N1 3 OR AB Herpes-Virus N1 3 |
| S7 | TI HHV3 OR AB HHV3 |
| S6 | TI HHV3 OR AB HHV3 |
| S5 | TI Shingles OR AB Shingles |
| S4 | TI "Herpesvirus 3" OR AB "Herpesvirus 3" |

##

## S1 Table. Risk of bias assessment for case series.

| **Studies** | **Evaluation *** | | | | | | | | | |
| --- | --- | --- | --- | --- | --- | --- | --- | --- | --- | --- |
|  | **1** | **2** | **3** | **4** | **5** | **6** | **7** | **8** | **9** | **Final** |
| Álvarez 2007 [1] | Yes | Yes | Yes | NA | NA | Yes | NR | Yes | Yes | Good |
| Andrade 2019 [2] | Yes | Yes | Yes | NA | NA | Yes | NA | Yes | Yes | Good |
| Antoniolli 2019 [3] | Yes | Yes | Yes | NA | NA | Yes | Yes | Yes | Yes | Good |
| Bollea-Garlatti 2017 [4] | Yes | Yes | Yes | Yes | NA | No | Yes | Yes | Yes | Good |
| Corti 2015 [5] | Yes | Yes | Yes | NA | NA | Yes | NA | Yes | Yes | Good |
| de Martino Mota  2016 [6] | Yes | Yes | Yes | NA | NA | Yes | Yes | Yes | Yes | Good |
| González 2013 [7] | Yes | Yes | Yes | NA | NA | Yes | Yes | No | Yes | Good |
| Gormezano 2015 [8] | Yes | No | ND | NA | NA | Yes | NR | Yes | Yes | Poor |
| Mendoza Rodríguez  2007 [9] | Yes | Yes | Yes | NA | NA | Yes | NA | Yes | Yes | Good |
| Rozenek 2018 [10] | Yes | Yes | Yes | NA | NA | NR | Yes | Yes | No | Good |
| Rueda 2010 [11] | Yes | Yes | No | NA | NA | Yes | Yes | Yes | Yes | Good |
| Teive 2008 [12] | Yes | Yes | Yes | NA | NA | Yes | NA | Yes | No | Good |
| Vázquez 2017 [13] | Yes | No | Yes | NA | NA | Yes | NA | Yes | Yes | Good |
| Vujacich 2008 [14] | Yes | Yes | Yes | NA | NA | Yes | NA | Yes | Yes | Good |
| Wagerman 2014 [15] | Yes | Yes | Yes | NA | NA | Yes | NA | Yes | Yes | Good |

* NA: Not applicable, NR: Not reported, ND: Not determined

1. Was the study question or objective clearly specified?
2. Was the study population clearly and fully described, including case definition?
3. Were cases consecutive?
4. Were subjects comparable?
5. Was exposure clearly described?
6. Were measures of results clearly defined, valid, reliable and consistently implemented for all study participants?
7. Was the length of follow-up appropriate?
8. Were statistical methods properly described?
9. Were results properly described?

## S2 Table. Risk of bias assessment for cohort studies.

| **Studies** | **Evaluation *** | | | | | | | | | | | | | | | |
| --- | --- | --- | --- | --- | --- | --- | --- | --- | --- | --- | --- | --- | --- | --- | --- | --- |
|  | **1** | **2** | **3** | **4** | **5** | **6** | **7** | **8** | **9** | **10** | **11** | **12** | **13** | **14** | **Final** |  |
| Alarcón 2014 [16] | Yes | Yes | Yes | Yes | No | Yes | Yes | NA | No | NA | No | NA | Yes | NR | Good |  |
| Borba 2010 [17] | Yes | Yes | Yes | Yes | No | Yes | Yes | NA | Yes | NA | Yes | NA | Yes | NR | Good |  |
| Carvalho 2016 [18] | Yes | Yes | Yes | Yes | No | Yes | Yes | NA | Yes | NA | ND | No | ND | Yes | Good |  |
| Castañeda 2017 [19] | Yes | Yes | ND | No | No | Yes | Yes | No | No | ND | NO | NA | NR | NR | Bad |  |
| Cortés 2008 [20] | Yes | Yes | ND | Yes | No | Yes | Yes | Yes | Yes | Yes | Yes | NA | NR | No | Good |  |
| Kawai 2015 [21] | Yes | Yes | Yes | ND | No | Yes | Yes | NA | Yes | NA | Yes | NA | ND | Yes | Good |  |
| Rampakakis 2017 [22] | Yes | Yes | NR | Yes | No | Yes | Yes | NA | Yes | Yes | Yes | NA | No | No | Good |  |
| Rampakakis 2019 [23] | Yes | No | NR | NR | NR | Yes | Yes | NA | ND | NA | Yes | NA | NR | NR | Poor |  |
| Vujacich 2016 [24] | Yes | Yes | NR | Yes | No | Yes | Yes | NA | Yes | Yes | Yes | NA | Yes | NR | Good |  |
| Zerbini 2016 [25] | No | Yes | NR | NR | NR | Yes | ND | ND | No | NA | Yes | NA | NR | Yes | Bad |  |
| Toniolo-Neto 2018 [26] | Yes | Yes | NR | Yes | No | Yes | Yes | NA | Yes | Yes | Yes | NA | Yes | No | Good |  |

* NA: Not applicable, NR: Not reported, ND: Not determined

1. Was the study question or research objective clearly specified?

2. Was the study population clearly specified and defined?

3. Did at least 50% of eligible subjects take part?

4. Were all subjects screened or recruited from the same population or from similar populations (including the same period of time)? Were inclusion and exclusion criteria to take part in the study pre-specified and applied consistently to all participants?

5. Was rationale for sample size, power description or variance and effect estimations provided?

6. For analysis of this study, were the exposures of interest measured before results?

7. Was the follow-up period enough for one to reasonably expect to observe an association between exposure and result, if any?

8. For exposures that may vary in terms of amount or level, did the study examine different levels of exposure relative to the result (e.g. categories of exposure or exposure measured as a continuous variable)?

9. Were measures of exposure (independent variables) clearly defined, valid, reliable and consistently implemented for all study participants?

10. Were exposures evaluated more than once over time?

11. Were measures of results (dependent variables) clearly defined, valid, reliable and consistently implemented for all study participants?

12. Were results raters blinded to participants’ exposure?

13. Were lost to follow-up 20% or less after the study startup?

14. Were potential confounding variables key due to their impact on the exposure(s)-result(s) ratio measured and statistically adjusted?

## S3 Table. Reported HZ complications and their treatment.

| **Reference** | **PHN (number)** | **Length of PHN in months (mean)±SD)** | **OHZ (number)** | **RHS (number)** | **Neurological complications (number)** | **Secondary bacterial infections (number)** | **DHZ (number)** | **Antivirals (number of individuals receiving treatment and specific type)** | **Steroids (number)** | **Pain management treatment (number and specific type)** | **Antibiotics (number)** | **Prophylaxis (number)** | **Other treatments (number and specific type)** |  |
| --- | --- | --- | --- | --- | --- | --- | --- | --- | --- | --- | --- | --- | --- | --- |
| Bollea-Garlatti 2017 [4] | 10 | NR | 7 | NR | Meningoencephalitis 2 | 15 | 41 | **41** Acyclovir PO (35) ** Acyclovir IV (41) ** | NR | NR | NR | NR | NR |  |
| Corti 2015 [5] | NR | NR | NR | NR | Meningoencephalitis 11 | NR | NR | **11** Acyclovir EV (11) | NR | NR | NR | NR | NR |  |
| Rozenek 2018[10] | 199** | NR | NR | NR | NR | NR | NR | 1,177 | NR | **133** NSAIDs (97) Pregabalin (86) | NR | NR | NR |  |
| Vujacich 2008 [14] | 39 | NR | 6 | NR | NR | 15 | 23 | **271** Acyclovir PO (159) Valacyclovir PO (78) Famciclovir PO (3) Acyclovir IV (1) Foscarnet IV (1) | 39 | **205** NSAIDs (94) Amitriptyline/ Carbamazepine (48) Opioids (30) | NR | NR | **41** Vitamin B (40) Topical acyclovir (24) Alternative therapies (5) |  |
| Vujacich 2016 [27] | 11 | NR | NR | NR | NR | NR | NR | **87** Acyclovir PO (49) Valacyclovir PO (32) Famciclovir PO (9) | 3 | **127** NSAIDs (74) Anti-epileptic drugs (17) Opioids (15) Antidepressants (9) Topical medication (12) | 5 | NR | 5 Ophthalmic drugs (3) Anxiolytics (2) |  |
| Antoniolli 2019 [3] | 178 | NR | 61 | 14 | Meningoencephalitis  22 | NR | 129 | **760** Acyclovir PO (320) Acyclovir IV (440) | NR | NR | NR | NR | NR |  |
| Álvarez 2007 [1] | 18 | 52.9 | NR | 1 | NR | NR | NR | NR | NR | Amitriptyline (9) Chlorpromazine (6) Gabapentin (4) Carbamazepine (3) Imipramine (2) | NR | NR | NR |  |
| Andrade 2019 [2] | NR | NR | 19 | NR | NR | NR | NR | **19** Acyclovir PO (17) Valacyclovir PO (2) | NR | NR | NR | NR | NR |  |
| Borba 2010 [17] | 10 | NR | 0 | NR | NR | 6 | 0 | **51** Acyclovir PO (46) Acyclovir EV (5) | NR | **51** NSAIDs (51) | NR | NR | NR |  |
| Gormezano 2015 [28] | 16 | NR | 1 | NR | NR | 9 | 1 | **19** Acyclovir EV (19) | NR | NR | NR | NR | NR |  |
| Toniolo-Neto 2018 [26] | 72 | NR | NR | NR | NR | NR | NR | **40** Acyclovir PO (33) Valacyclovir PO (8) | 9 | **99** Analgesics (50) (Acetaminophen (23) AAS (1) Metamizole (19), Hyoscine (2), Others (16)) Anticonvulsants (13) Antidepressants (13) Opioids (12) | NR | NR | **114** Topical treatments (31) Anti-inflammatory drugs (5) Benzodiazepines (3) Antihypertensive drugs (5) Vitamins (5) Others (65) |  |
| Rampakakis 2019 [29] | 56 | NR | NR | NR | NR | NR | NR | 148** | NR | 139** | NR | NR | NR |  |
| Rampakakis 2017 [22] | 40 | NR | NR | NR | NR | NR | NR | **31** Acyclovir PO (27) Valacyclovir PO (4) Famciclovir PO (2) | 9 | **46** NSAIDs (7) Acetaminophen (12) Anti-epileptic drugs (13) Opioids (11) Antidepressants (3) | NR | NR | **16** Topical treatments (antivirals, antibiotics, and steroids) (16) Anxiolytics (3) |  |
| González 2013 [30] | 14 | NR | 19 | NR | Motor paralysis  2 | NR | NR | **12** Acyclovir PO (12) | 9 | 19 | NR | NR | Ophthalmic drugs |  |
| Vázquez 2017 [13] | 775 | NR | 493 | NR | Meningoencephalitis  380 | NR | 197 | NR | NR | NR | NR | NR | NR |  |
| Mendoza Rodríguez 2007 [9] | 134 | NR | NR | NR | NR | 46 | 3 | **261** Acyclovir (184) Valacyclovir PO (60) Brivudine PO (17) Isoprinosine (4) | 2 | **102** Analgesics (71) Tricyclic antidepressants (31) | 9 | NR | **535** Topical drying agents (445) Topical analgesics (1) Topical antivirals (10) Topical antibiotics (4) 2 or more topical drugs (63) Neurotropic vitamins (12) |  |
| DHZ: disseminated herpes zoster, IV: intravenous injection, OHZ: ocular herpes zoster, PO: prescribed orally, PHN: post-herpetic neuralgia, SD: standard deviation, RHS: Ramsay Hunt syndrome, NSAID: nonsteroidal anti-inflammatory drug, NR: not reported | | | | | | | | | | | | | | |
| **Additional data provided by the author | | | | | | | | | | | | | | |

## S4 Table. Chile: Average length of stay per ICD-10 category per year.

| **Condition** | **Code ICD-10** | **2010** | **2011** | **2012** | **2013** | **2014** | **2015** | **2016** | **2017** | **2018** | **Average** |
| --- | --- | --- | --- | --- | --- | --- | --- | --- | --- | --- | --- |
| Encephalitis caused by HZ | B020 | 9 | 7.5 | 6.33 | 19.91 | 16.67 | 16 | 17.61 | 19.91 | 17.54 | 14.50 |
| Meningitis caused by HZ | B021 | 5.9 | 6.12 | 9.33 | 10.14 | 6.62 | 8 | 16.86 | 8.58 | 8.61 | 8.91 |
| HZ with CNS involvement | B022 | 6.62 | 5.09 | 4.63 | 5.42 | 6.13 | 6.75 | 6.48 | 9.26 | 6.7 | 6.34 |
| Ocular HZ | B023 | 6.48 | 6.5 | 5.94 | 6.97 | 6.05 | 8.82 | 8.06 | 6.47 | 6.74 | 6.89 |
| Disseminated HZ | B027 | 8.75 | 8.71 | 8.53 | 7.94 | 9.61 | 7.67 | 7.37 | 8.32 | 7.61 | 8.28 |
| HZ with other complications | B028 | 5.9 | 5.78 | 5.88 | 5.83 | 6.44 | 6.87 | 6.9 | 6.19 | 5.71 | 6.17 |
| Uncomplicated HZ | B029 | 6.3 | 6.86 | 5.76 | 6.66 | 6.93 | 5.35 | 6.17 | 7.33 | 5.96 | 6.37 |
| PHN | G530 | NA | NA | NA | NA | 6.33 | 3.5 | NA | NA | 1 | 3.61 |

ICD-10: International Classification of Diseases, Tenth Revision, CNS: central nervous system, HZ: herpes zoster, PHN: post-herpetic neuralgia

## S5 Table. Chile: Case-fatality per ICD-10 category per year and on average in 2010-2018.

| **Condition** | **Code ICD-10** | **2010** | **2011** | **2012** | **2013** | **2014** | **2015** | **2016** | **2017** | **2018** | **Average** |
| --- | --- | --- | --- | --- | --- | --- | --- | --- | --- | --- | --- |
| Encephalitis caused by HZ | B020 | 0.00% | 0.00% | 0.00% | 8.33% | 0.00% | 0.00% | 0.00% | 0.00% | 0.00% | **0.93%** |
| Meningitis caused by HZ | B021 | 0.00% | 0.00% | 0.00% | 0.00% | 0.00% | 11.11% | 0.00% | 0.00% | 0.00% | **1.23%** |
| HZ with CNS involvement | B022 | 0.00% | 0.00% | 0.00% | 0.00% | 0.00% | 0.00% | 0.00% | 0.00% | 3.92% | **0.44%** |
| Ocular HZ | B023 | 0.00% | 0.00% | 0.00% | 0.00% | 0.00% | 0.00% | 0.00% | 0.00% | 0.00% | **0.00%** |
| Disseminated HZ | B027 | 0.00% | 7.14% | 0.00% | 0.00% | 0.00% | 7.41% | 3.70% | 3.23% | 0.00% | **2.39%** |
| HZ with other complications | B028 | 2.33% | 0.00% | 0.00% | 0.00% | 1.85% | 0.00% | 1.61% | 0.00% | 3.03% | **0.98%** |
| Uncomplicated HZ | B029 | 0.37% | 0.36% | 0.00% | 0.00% | 0.40% | 0.00% | 1.49% | 0.00% | 0.00% | **0.29%** |
| PHN | G530 | N/C | N/C | 0.00% | N/C |  | 0.00% | N/C | N/C | 0.00% | **0.00%** |
| Total |  | **0.46%** | **0.45%** | **0.00%** | **0.23%** | **0.45%** | **0.72%** | **1.34%** | **0.21%** | **0.81%** | **0.52%** |

ICD-10: International Classification of Diseases, Tenth Revision, CNS: central nervous system, HZ: herpes zoster, PHN: post-herpetic neuralgia

## S6 Table. Chile: Total HZ deaths by ICD-10 categories reported by the Ministry of Health, 2010-2017.

| **Condition** | **Code ICD-10** | **2010** | **2011** | **2012** | **20013** | **2014** | **2015** | **2016** | **2017** | **Total** |
| --- | --- | --- | --- | --- | --- | --- | --- | --- | --- | --- |
| Encephalitis caused by HZ | B020 | 0 | 0 | 1 | 0 | 0 | 0 | 0 | 2 | **3** |
| Meningitis caused by HZ | B021 | 1 | 0 | 0 | 0 | 0 | 0 | 0 | 0 | **1** |
| HZ with CNS involvement | B022 | 0 | 0 | 0 | 0 | 0 | 1 | 0 | 0 | **1** |
| Ocular HZ | B023 | 0 | 0 | 0 | 0 | 0 | 0 | 1 | 0 | **1** |
| Disseminated HZ | B027 | 0 | 0 | 1 | 0 | 0 | 1 | 0 | 0 | **2** |
| HZ with other complications | B028 | 0 | 0 | 0 | 0 | 0 | 0 | 0 | 1 | **1** |
| Uncomplicated HZ | B029 | 0 | 4 | 3 | 5 | 1 | 5 | 2 | 2 | **22** |
| Total |  | **0** | **4** | **5** | **5** | **1** | **7** | **3** | **5** | **31** |

ICD-10: International Classification of Diseases, Tenth Revision, CNS: central nervous system, HZ: herpes zoster, PHN: post-herpetic neuralgia

## S1 Fig. Mexico: Average hospital discharges per ICD-10 category in 2010-2017.

ICD-10: International Classification of Diseases, Tenth Revision

## S2 Fig. Mexico: Average length of stay in hospital per ICD-10 category in 2010-2017.

ICD-10: International Classification of Diseases, Tenth Revision

## S3 Fig. Mexico: Case-fatality rate per year.

## S4 Fig. Mexico: Total deaths in 2010-2017.

## S5 Fig. Chile: Hospital discharges per year per ICD-10 category 2010-2018.


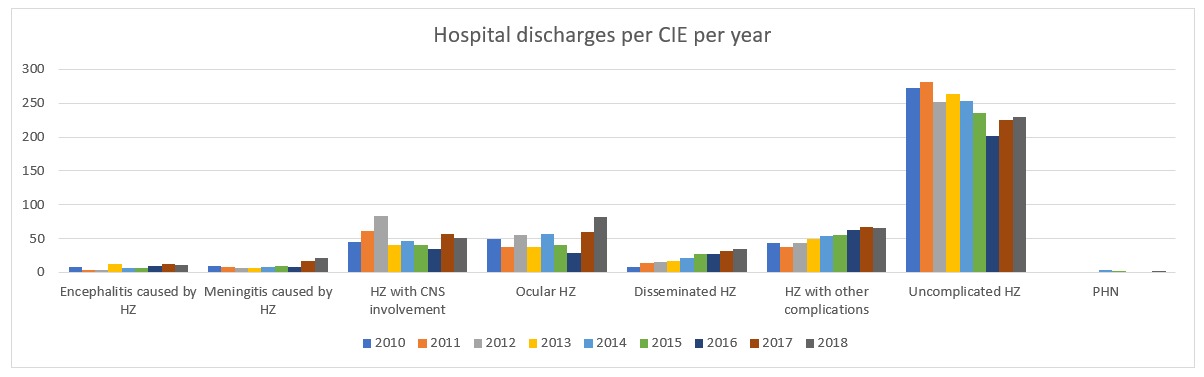


ICD-10: International Classification of Diseases, Tenth Revision

## S6 Fig. Chile: Average of average annual stays in hospital per ICD-10 category.

ICD-10: International Classification of Diseases, Tenth Revision

# Focus on the patient

## What is the context?

- Herpes Zoster (HZ) or shingles, is caused by reactivation of varicella zoster virus which causes chickenpox in childhood.
- Older individuals (>50 years of age) and those with other conditions (immune system dysfunction) are high-risk groups for acquiring HZ. Improper functioning of the immune system in adults also adds to the risk of HZ disease and its complications.
- Disease burden of HZ is largely unknown for the Latin America and Caribbean (LAC) region.

## What is new?

- This review summarizes findings from 26 studies in LAC.
- No studies reported the epidemiology and burden of HZ in the general population.
- High incidence and death rates were observed in high-risk populations; disease incidence, severity and the occurrence of complications were mainly driven by the age of the individual.
- Common HZ complications were post-herpetic neuralgia, ophthalmic herpes zoster, and Ramsay Hunt syndrome. Complications were higher in the immunosuppressed compared to the immunocompetent population.

## What is the impact?

- The study provides a comprehensive overview on HZ epidemiology and burden in a region where very limited information is available due to lack of mandatory reporting.
- High-risk groups, such as older individuals and those with immune system dysfunctions, are vulnerable to HZ and its complications. These individuals could benefit from directed healthcare initiatives.
- This study shows that overall HZ occurrence in the general population of LAC was low but increased with an increase in age of the individual or in individuals with immune

***References***

1. Alvarez FK, de Siqueira SR, Okada M, Teixeira MJ, de Siqueira JT. Evaluation of the sensation in patients with trigeminal post-herpetic neuralgia. J Oral Pathol Med. 2007;36(6):347-50. Epub 2007/06/15. doi: 10.1111/j.1600-0714.2006.00489.x. PubMed PMID: 17559496.

2. Andrade FMX, Bezerra FM, Santos MSD, Araujo MEXdS. Clinical profile and ophthalmologic manifestations of Herpes Zoster Ophthalmicus. Revista Brasileira de Oftalmologia. 2019;78:170-4.

3. Antoniolli L, Rodrigues C, Borges R, Goldani LZ. Epidemiology and clinical characteristics of herpes zoster in a tertiary care hospital in Brazil. Braz J Infect Dis. 2019;23(2):143-5. Epub 2019/04/03. doi: 10.1016/j.bjid.2019.03.001. PubMed PMID: 30935817.

4. Bollea-Garlatti ML, Bollea-Garlatti LA, Vacas AS, Torre AC, Kowalczuk AM, Galimberti RL, et al. Clinical Characteristics and Outcomes in a Population With Disseminated Herpes Zoster: A Retrospective Cohort Study. Actas Dermo-Sifiliográficas (English Edition). 2017;108(2):145-52. doi: <https://doi.org/10.1016/j.adengl.2016.12.019>.

5. Corti M, Villafañe MF, Vittar N, Banco MC, Priarone M, Mammana L, et al. Meningoencephalitis due to varicella zoster virus in aids patients. Report of eleven cases and review of the literature. Rev Inst Med Trop Sao Paulo. 2015;57(6):505-8. doi: 10.1590/S0036-46652015000600007. PubMed PMID: 27049704.

6. de Martino Mota A, Carvalho-Costa FA. Varicella zoster virus related deaths and hospitalizations before the introduction of universal vaccination with the tetraviral vaccine. J Pediatr (Rio J). 2016;92(4):361-6. Epub 2016/03/13. doi: 10.1016/j.jped.2015.10.003. PubMed PMID: 26969400.

7. González KG, Alonzo-Romero PL, Campos AG. Herpes zoster oftálmico. Evaluación de complicaciones y secuelas oculares y su relación con diversos tratamientos. Dermatologia Revista Mexicana. 2013;56:392-8.

8. Gormezano NWS, Silva CA, Otsuzi CI, Barros DL, da Silva MA, Sallum AME, et al. Higher Prevalence and Distinct Features of Herpes Zoster Infection in Children than Adults with Systemic Lupus Erythematosus. Pediatr Infect Dis J. 2015;34(8):905-7. doi: 10.1097/inf.0000000000000756. PubMed PMID: 26020409.

9. F.I. MR, C.J. TB. Comportamiento clínico y terapéutico del herpes zoster en el centro nacional de dermatología “dr. Francisco josé gómez urcuyo” enero 2002 - Diciembre 2006. Managua: s.n; 2007.

10. Rozenek M, Boietti B, Romani A, Ramilo M, Cámera L. Herpes zoster and Post herpetic neuralgia in elderly adults in a community hospital in Buenos Aires. June 2013-May 2017. International Journal of Infectious Diseases. 2018;73:366-7. doi: 10.1016/j.ijid.2018.04.4244.

11. Rueda M. Variación estacional en el Herpes zoster^ies Seasonal variation in herpes zoster^ien. Dermatol peru. 2010;20(2):99-106.

12. Teive HA, Funke V, Bitencourt MA, de Oliveira MM, Bonfim C, Zanis-Neto J, et al. Neurological complications of hematopoietic stem cell transplantation (HSCT): a retrospective study in a HSCT center in Brazil. Arquivos de neuro-psiquiatria. 2008;66(3b):685-90. doi: 10.1590/s0004-282x2008000500014.

13. Vazquez M, Cravioto P, Galvan F, Guarneros D, Pastor VH. [Varicella and herpes zoster: challenges for public health]. Salud Publica Mex. 2017;59(6):650-6. Epub 2018/02/17. doi: 10.21149/7997. PubMed PMID: 29451634.

14. Vujacich C, Poggi E, Cecchini D, Luchetti P, Stamboulian D. [Clinical and epidemiological aspects of herpes zoster]. Medicina (B Aires). 2008;68(2):125-8. Epub 2008/05/27. PubMed PMID: 18499960.

15. Wagemann B R WBH, Wagemann B E, Huerta R J, Wagemann F R, Wagemann H P. Dermatosis del adulto mayor en Antofagasta, experiencia de 30 años. Rev chil dermatol. 2014.

16. Alarcón ML, Esper JA, Alzate F, Higuera SA, Fajardo JE, Solier Insuasty J. Asociación entre herpes zóster y recaída o progresión de neoplasias sólidas. Acta Medica Colombiana. 2014;39:35-9.

17. Borba EF, Ribeiro AC, Martin P, Costa LP, Guedes LK, Bonfa E. Incidence, risk factors, and outcome of Herpes zoster in systemic lupus erythematosus. Journal of clinical rheumatology : practical reports on rheumatic & musculoskeletal diseases. 2010;16(3):119-22. doi: 10.1097/RHU.0b013e3181d52ed7.

18. Carvalho H, Laurindo IMM, Ranza R, Titton D, Bertolo M, Bianchi W, et al. Herpes zoster in the Brazilian register-biobadabrasil. Annals of the Rheumatic Diseases. 2016;75:881-2. doi: 10.1136/annrheumdis-2016-eular.5238.

19. Castañeda OM, Romero FJ, Salinas A, Citera G, Mysler E, Rillo O, et al. Safety of Tofacitinib in the Treatment of Rheumatoid Arthritis in Latin America Compared With the Rest of the World Population. JCR: Journal of Clinical Rheumatology. 2017;23(4):193-9. doi: 10.1097/rhu.0000000000000498. PubMed PMID: 00124743-201706000-00003.

20. Cortés C, Beltrán C, Muñoz R, Daube E, Wolff M. [Impact of baseline CD4 count, immune recovery and viral suppression at 7 year of first highly active antiretroviral therapy on survival, AIDS defining events and immune recovery reactions]. Rev Med Chil. 2008;136(12):1503-10. Epub 2009/04/08. PubMed PMID: 19350166.

21. Kawai K, Rampakakis E, Tsai TF, Cheong HJ, Dhitavat J, Covarrubias AO, et al. Predictors of postherpetic neuralgia in patients with herpes zoster: a pooled analysis of prospective cohort studies from North and Latin America and Asia. International journal of infectious diseases : IJID : official publication of the International Society for Infectious Diseases. 2015;34:126-31. doi: 10.1016/j.ijid.2015.03.022.

22. Rampakakis E, Alpizar C, Karellis A, Sampalis JS, Johnson K, Monsanto HA, et al. Measuring the burden of herpes zoster disease in Costa Rica. Acta Médica Costarricense. 2017;59:146-52.

23. Rampakakis E, Monsanto HA, Stutz M, Psaradellis E, Mejia G, Carillo AE, et al. Pin18 Burden of Illness of Herpes Zoster in Colombia: An Observational Study. Value in Health Regional Issues. 2019;19. doi: 10.1016/j.vhri.2019.08.249.

24. Vujacich C, de Wouters L, Margari AM, Gordóvil M, Rampakakis E, Psaradellis E, et al. Dolor, calidad de vida relacionada con la salud y utilización de servicios médicos asociados al herpes zoster en Argentina. Actualizaciones en SIDA e Infectología. 2016;24(92):53-63.

25. Zerbini CAF, Radominski SC, Cardiel M, Castañeda O, Citera G, Neira O, et al. Herpes zoster in patients with rheumatoid arthritis: Pooledanalyses of to facitinib phase 2, 3 and long-term extension studies in a Latin American subpopulation. Journal of Clinical Rheumatology. 2016;22(3):124. doi: 10.1097/RHU.0000000000000372.

26. Toniolo-Neto J, Psaradellis E, Karellis A, Rampakakis E, Rockett TY, Sampalis JS, et al. Measuring herpes zoster disease burden in São Paulo, Brazil: a clinico-epidemiological single-center study. Clinics (Sao Paulo). 2018;73:e243-e. doi: 10.6061/clinics/2018/e243. PubMed PMID: 30043824.

27. Vujacich C dWL, Margari AM, et al. Dolor,. calidad de vida relacionada con la salud y utilización de servicios médicos asociados al herpes

zoster en Argentina. 2016.

28. Gormezano NW, Silva CA, Otsuzi CI, Barros DL, da Silva MA, Sallum AM, et al. Higher Prevalence and Distinct Features of Herpes Zoster Infection in Children than Adults with Systemic Lupus Erythematosus. Pediatr Infect Dis J. 2015;34(8):905-7. doi: 10.1097/inf.0000000000000756.

29. Rampakakis E, Monsanto HA, Stutz M, Psaradellis E, Mejia G, Carillo AE, et al. Burden of illness of herpes zoster in colombia: An observational study. Value in Health Regional Issues. 2019;19:S43. doi: 10.1016/j.vhri.2019.08.249.

30. González KG, Alonzo-Romero PL, Campos AG. Herpes Zoster oftálmico. Evaluación de complicaciones y secuelas oculares y su relación con diversos tratamientos. Dermatologia Revista Mexicana. 2013;56(6):392-8.
