# Supplementary material for: Herpes zoster epidemiology in Latin America: A systematic review and meta-analysis
Source: PLoS One. 2021 Aug 12;16(8):e0255877. doi: 10.1371/journal.pone.0255877 (PMC8360515; doi:10.1371/journal.pone.0255877)
Supplement: Attachment — Submitted filename: response letter.docx [file pone.0255877.s003.docx]

Reviewer #1: Very well written. I would agree that it is important to evaluate HZ in different populations, and this provides rationale for the implementation of a vaccine program in the LAC region. One thing I would like to see added is mention on if the patients in any of the studies were vaccinated? 
**Thank you. None of the 23 included studies mention the vaccination status after carefully checking this.   A line was inserted in the results describing this important fact.** 
 
Reviewer #2: The paper addresses a relevant and interesting topic such as the evaluation of HZ epidemiology in Latin America. 
The manuscript is interesting, well written and quite complete. 
There are few points that deserve a comment: 
- crude numbers have been included in some parts of the manuscript; it should be better to have rates (e.g. lines 223, 225, 228) 
**Thank you, we re-wrote this into frequencies.**  
 
- deaths and CFR are extensively taken into account. Authors correctly point out that these data should be considered with caution as many deaths could occur in patients with co-morbidities/high risk and could not be directly attributed to HZ. For this reason it seems that these data are described in too much detail 
**Thank you. The authors’ group feel no change is needed in this regard, as the cautionary note is included.** 
 
- the lenght of stay seems quite short in respect to what have been registered elsewhere. For example, a survey in Italy on HZ hospitalized subjects pointed out an average lenght of stay equal to 23 days. This reference could be added as well as discussed (Valente N et al: Temporal trends in herpes zoster-related hospitalizations in Italy, 2001-2013: differences between regions that have or have not implemented varicella vaccination. Aging Clin Exp Res DOI 10.10'7/s40520-017-0782-z) 
**Thank you. We added this contrast in the discussion.** 
 
 
It could be useful and interesting to know if Authors have evaluated or considered a changed trend in HZ incidence in relation to the eventual use of universal varicella vaccination in the considered countries. This is a relevant and amply debated issue and it could be interesting to have this point included at least in the discussion. 
Besides, it could be interesting to know if any introduction of HZ vaccination is foreseen in the considered countries and with type of HZ vaccine. 
 
**Thank you. We added two references regarding this important aspect. Arlant 2019 BMC PH, and Cashat et al 6th ISPOR Latam Conference.  (Refs in the paper)**
